# Co-Produced Care in Veterinary Services: A Qualitative Study of UK Stakeholders’ Perspectives

**DOI:** 10.3390/vetsci7040149

**Published:** 2020-10-01

**Authors:** Alison Z. Pyatt, Keith Walley, Gillian H. Wright, Emma C. L. Bleach

**Affiliations:** 1 Equine Department, Hartpury University, Hartpury, Gloucester GL19 3BE, UK; 2 Department of Animal Production, Welfare and Veterinary Sciences, Harper Adams University, Newport TF10 8NB, UK; kwalley@harper-adams.ac.uk (K.W.); gillianhwright@btinternet.com (G.H.W.); ebleach@harper-adams.ac.uk (E.C.L.B.)

**Keywords:** veterinary service, co-production, veterinary clients, veterinary communication, qualitative, grounded theory

## Abstract

Changes in client behaviour and expectations, and a dynamic business landscape, amplify the already complex nature of veterinary and animal health service provision. Drawing on prior experiences, veterinary clients increasingly pursue enhanced involvement in services and have expectations of relationship-centred care. Co-production as a conceptualisation of reciprocity in service provision is a fundamental offering in the services sector, including human medicine, yet the role of co-production in veterinary services has been minimally explored. Utilising a service satisfaction framework, semi-structured interviews (*n* = 13) were completed with three veterinary stakeholder groups, veterinarians, allied animal health practitioners, and veterinary clients. Interview transcript data were subject to the qualitative data analysis techniques, thematic analysis and grounded theory, to explore relationship-centred care and subsequently conceptualise co-production service for the sector. Six latent dimensions of service were emergent, defined as: empathy, bespoke care, professional integrity, value for money, confident relationships, and accessibility. The dimensions strongly advocate wider sector adoption of a co-produced service, and a contextualised co-production framework is presented. Pragmatic challenges associated with integration of active veterinary clients in a practitioner–client partnership are evident. However, adopting a people-centric approach to veterinary services and partnerships with clients can confer the advantages of improved client satisfaction, enhanced treatment adherence and outcomes, and business sustainability.

## 1. Introduction

Undeniably, the veterinary business landscape is experiencing a period of unprecedented change. Corporate consolidation and a growth in practice size [1], digitisation and telemedicine [2,3], the rise of pet care services [4], attrition rate of veterinarians, job dissatisfaction and burnout [5], and the feminisation of the profession [6,7,8] all contribute to the transformation of the sector. Across the allied animal health sector (paraprofessional practitioners), the specialist services offered continue to grow and develop in all areas of animal health [1,8].

Concomitant changes are evident in client behaviour and client expectations of veterinary services. Animal owners and keepers are more discerning and sophisticated than ever before. They are arguably more knowledgeable, and have constant access to readily available information, data, and knowledge through on-line sources and search engines [9,10]. Through social media platforms, clients connect with other like-minded animal owners and share experiences and opinions rapidly through positive and negative word-of-mouth communication. Clients can select veterinary or animal health services quickly and easily, and veterinary practice loyalty is now an outdated behaviour [11]. 

Client relationships with the animals they keep has been transformative. Anthropomorphised pets are considered to be a family member [12,13,14] and clients hold expectations of health care parallel to human services. Drawing on personal experience of health services, clients have expectations of equivalence in the veterinary services received, and consequently service expectations continue to rise [1,8].

The developing perspective of social licence adds weight to the client expectation of veterinary health provision. For sporting, performance, or production animal enterprises, such as the horse industry and agriculture, conceptualisation of social licence is a rapidly developing framework. Social licence or a Social Licence to Operate (SLO), has been defined as an “intangible, unwritten and non-legally binding social contact” [15] for those seeking legitimacy for their practice, or in this context, their use of animals. Contextualised for human–animal relationships, basic adherence to animal welfare legislation or farm animal assurance schemes is no longer considered as sufficient to secure broad public acceptance. The concept of social licence is increasingly realised in sporting equestrian pursuits [16] and livestock production [17,18]. 

Until the time of the Vet Futures Project in 2015 [19], the UK animal health sector had paid minimal attention to client-centric service [6]; accordingly there is a paucity of client service provision literature. There is an evolving body of research to support the role of veterinarian–client communication in client satisfaction and patient outcome [20,21,22], the findings of which confirm the relevance of trust and empathy [23] to relationship-centred care. The strong evidence base in communication skills is patent [21,22], but comprehension on working with veterinary clients in a relational co-produced partnership, equivalent to human health provision, has been minimally explored. Commentary in the veterinary sector has identified the provision of relationship-centred services to be an area requiring improvement to reduce the risk of litigation [24], to meet rising client expectations, and as a business opportunity [6,20], and is proposed to serve as a strategy to improve the working environment for the veterinarian. Recognition that veterinary services should be providing an inherently relationship-centred approach is evident [20,21,22,23]. However, co-produced services necessitate a different and distinctive approach.

Co-production, or relationship-focused service, has its origins in 1970s services marketing, defining the transformation of the goods versus services marketing construct through to recognition of the intrinsic role of the client or customer [25]. Co-production is now embedded in education [26], governance, and the public sector [27,28], and was fundamental to the reform of the UK National Health Service (NHS) [29]. At the NHS policy level, service–user collaboration through co-production is an accepted requirement [30] and patient-centred care a reality [31]. At its heart, co-production is value-driven, and centred in reciprocity and mutuality: it is inherently founded in relationship-centric behaviours. Extended to human health service provision, co-production operates to deliver services in an equal and reciprocal relationship through the establishment of patient partnerships. Co-production recognises people as assets, thereby enhancing reciprocity and relationship development whilst engaging deeply in active dialogue with clients [32,33]. Human health providers face extreme challenges due to the inherent complexity of disease and illness, which manifest in different ways within each unique patient and situation [34]. Decisions regarding treatment may vary from patient to patient even when working to cure the same disease, and patterns of working are not dissimilar to veterinary care. Health service is, therefore, highly complex, intangible, and intrinsically based on co-production. 

Research in human health services is most commonly focused on the recovery of wellness and the alleviation of suffering. These are essential processes which have explicit outcomes and the results achieved are tangible. Intangible aspects of health service, such as caregiving, are fundamentally important to the patient and the patient experience but are difficult to achieve due to the uniqueness of each relationship, combined with pressures of time and available finance. To achieve high-quality patient care, the service must be highly customised as each patient perceives and receives care in an individualised manner. Health providers have reflected on developing co-production systems of care which are “patient-centric” [35], recognising that improved patient experience within areas of communication [36], empathy, and perceived courtesy [37] result in greater levels of patient satisfaction. Patients seek holistic whole person care, which includes emotional support, and want the courtesy of being treated as an individual and with compassion [38]. 

The delivery of human health service has adapted from historically being provider-centred to patient-centred [34], a paradigm shift more recently mirrored by the veterinary sectors [19]. Parallels between human health service provision and that of veterinary care are evident, but to date, there has been limited enquiry into practitioner–client co-produced relations within the animal health sector. Within this present study, veterinary stakeholders (including veterinary service providers and clients) were questioned on topics of service provision and experience, including service satisfaction and dissatisfaction, and perceptions of service quality; with the aims of exploring the veterinary service encounter in the context of service co-production and to identify constituents of co-production in the UK veterinary and animal health sector. 

## 2. Materials and Methods

### 2.1. Study Design

A qualitative research approach was adopted to explore veterinary stakeholder opinion and experience of service received and given. The methodology was selected as a valuable technique to appreciate complex issues of attitudes, perceptions, and opinions [39], and to explore and generate knowledge based on human experience. It did not seek to measure or quantify co-produced service for the sector. Data were collected using semi-structured interviews with the three stakeholder groups categorised as veterinarians, allied animal health practitioners (herein referred to as allied practitioners), and clients. Identification and selection of participants was performed using homogeneous purposive sampling, ensuring a well-informed and germane contribution [40]. Participants were selected according to the following criteria: professional role (veterinarian or allied practitioner), or client, age, and species of animal kept or treated. Participants were geographically dispersed over the following countries of the United Kingdom: England, Scotland, and Wales. Three veterinarians, five allied practitioners and five clients were interviewed.

A summary of participating stakeholders is provided in Table 1. Recruitment of participants was achieved through professional, academic, and industry contacts. All subjects gave informed written consent for inclusion before they participated in the study. The study was conducted in accordance with the Declaration of Helsinki, and the protocol was approved by the Ethics Committee of Harper Adams University, UK (code 4295-201412STAFF). Qualitative reporting follows the Consolidated Criteria for Reporting Qualitative Research (COREQ) process and checklist [41]. Techniques to standardise interview behaviour are beneficial to the process and validity of qualitative techniques. Accordingly, an interview sheet and prompt questions were devised using the extant literature, industry data, and professional knowledge of the researcher, and implemented (Supplementary Materials). Interview questions were developed from scoping of industry data, and veterinary and services literature. Questions were mapped to the study aims and for each question, prompts and probing questions were determined. Similar questions were posed to professionals and clients alike, but the interview language was contextualised. Clients were questioned on their experiences with the veterinarian and the allied practitioner and were encouraged to openly discuss their expectations of value and service satisfaction. Professionals discussed the service they provide and their perceptions of service quality and value. Pilot interviews were completed with a veterinarian, allied practitioner, and a client to ensure that the questions and terminology used were appropriate for each group. Data collected from the pilot study were rich, detailed, and relevant to the study, and were therefore analysed and included in the presented results. All participants spoke freely and were willing to share positive and negative personal experiences of service provided or received.

### 2.2. Data Collection

Over a 6-month period (July–December 2017), semi-structured face-to-face interviews (*n* = 13) were completed at either the participants place of work, home, or the primary researcher’s workspace. All interviews were completed by one researcher (A.Z.P.). Participants were attentive to the study aims and recruitment was uncomplicated. All interviews were audio-recorded using a SONY IC Recorder and stored as digital audio files (MP3) in accordance with UK general data protection regulations (GDPR). Interviewees were asked to share their experiences, beliefs, and opinions around a range of topics on animal health service provision, and the critical incident technique was used to encourage respondents to draw on past personal experiences and to aid recollection. At the culmination of each interview, all participants were given the opportunity to clarify any comments or to make further comment. Interview duration was between 45 and 92 min. 

### 2.3. Thematic Analysis

Theoretical thematic analysis, constructed in grounded theory methodology, with an iterative constant comparison technique, was used to identify common themes and patterns within the transcribed interview dataset. Thematic analysis and principles of grounded theory were selected for data analysis as a highly flexible approach, capable of yielding rich and detailed data on attitudes and experiences. The six-stage process, as defined by Braun and Clarke [42], was followed. Interviews were transcribed verbatim and re-read several times by the lead researcher (A.Z.P.) prior to the commencement of coding. Supplementary hand-written notes taken during each interview were used to further inform the research process. Thematic data analysis was primarily completed by A.Z.P. and was completed concurrent with data collection and transcription. Analysis was performed using the Qualitative Data Analysis software package QRS NVivo (v.11). The research team were core contributors to the initial code (theme) development or codebook through shared coding of the first pilot interview. Reliability was maximized through the use of memo-writing within the coding process, permitting transparency in the decision-making process of A.Z.P. and indicating to the research team how the data were interpreted. Research team validation was completed post-coding for each interview transcript. Embedded in grounded theory methodology [43], data saturation is reached when no new additional themes or theoretical codes are emergent [44]. In this study, data saturation was identified at interview number 12 and confirmed through the completion of an additional interview. 

## 3. Results

A sample of sector stakeholders reflecting age, gender, occupation, and species of animals kept or treated, was achieved, as shown in Table 1. Participants were considered to reflect the study population but are not generalisable. 

### 3.1. Thematic Analysis

The interview results disclosed six latent themes or dimensions within the data. The dimensions were defined as: Empathy, Bespoke care, Professional integrity, Value for money, Confident relationships, and Accessibility. These dimensions are described in Table 2. 

The category of emergent themes and explanatory narrative from the interview data are provided below.

#### 3.1.1. Theme One: Empathy

Dimensions of compassion, care, and empathy were discussed with all stakeholders and thoughtful communication was defined as an essential component of the interaction. All client groups, regardless of species kept, had expectations of considerate handling and treatment of their animals by all practitioners. Pet owners expressed this through the strength of bond between them and their pet.

One participant, on discussing their expectations of care and compassion in the veterinary consultation, expressed the emotional importance of their dog.

I think just that something that’s very precious to me is in their care and their understanding that I was feeling, that you are feeling anxious and worried and you want to know that everything is okay.

[07C]

Empathy towards the animal was found to be an expectation of the service provided, whereas empathy from practitioner to client was highly valued. In these cases, service delivery was emotive and highly charged as clients expressed the depth of compassion they had felt from the practitioners.

I remember her putting her arms around me and she was just really compassionate about how we were feeling.

[13C]

Equally, the strong person connection experienced was expressed even with a practitioner they had only just met.

She was lovely, I don’t know the vet’s name, it wasn’t the vet that I used to see on a regular basis for normal appointments and she was just really, really lovely.

[13C]

Factors of compassion and empathy were repeatedly, strongly, and overtly expressed within the allied practitioner group.

I think that you have got to have empathy and compassion with the animal.

[01P]

They also want to make sure that their pet is being cared for in the right way and they have got the best quality of care that there is, no matter what time of day. They want to see care and compassion in the situation.

[09P]

*I think they [*clients*] would be looking for a sympathetic hearing for what they want. I think they’d be looking for suitable amplification of the problems that they’re putting over, a solution to the problem that they’re presenting.*

[06P]

Veterinarians’ reflection of empathy was embedded within the service provision:

You’ve got to make them feel that their animals are important. The vet isn’t just looking at their watch and saying, “I’ve got another call to do”. The worst thing that vets can do is to say, “I’m in a hurry so I can’t be long at this”. It’s the while you’re here is the important thing and that welds the relationship between the client.

[03V]

#### 3.1.2. Theme Two: Bespoke Care

Bespoke care emerged as a strong theme, with the expectation that the service would be customised and individualised. Results and outcomes were important perceived components of a tailored package. Empathy and communication were anticipated to be important due to the inherent nature of health practice and were evident in the extant literature review.

They want expertise I think initially. They want attention when they want it, ASAP of course, especially in a crisis. You can understand that. They want latest information. They want expertise and they want practicality. They want pragmatism and they want understanding of their situation. There is a bespoke element to it. Although they wouldn’t voice it as that, there is that bespoke requirement—“I need this, and I need that”. The demands are high because they perceive the veterinarian as expensive.

[03V]

From the client perspective, there is a clear expectation for bespoke service to be delivered.

[In discussions with the farm vet] *After we’ve had the weekly routine [*visit*] they’ll always come up to the house. We’ll sit down and discuss things, if there’s an issue.*

[12C]

Interestingly, this time to talk with clients and provide individualised personal care was a fulfilling part of the practitioners’ role, suggestive of reciprocation in service delivery.

It’s not unusual to spend twenty minutes, half an hour, talking to somebody. I actually quite enjoy it.

[05P]

#### 3.1.3. Theme Three: Professional Integrity

Trust was patent within all participants’ interviews, but integral were notions of morality, integrity, and technical competence. Equally, the client expects the animal health professional to have the skill and ability to give the correct treatment well.

People aren’t going to trust your decision-making if they don’t think that you are a trustworthy person and that comes across in the way that you present yourself.

[04V]

Reciprocity in trust between the client and professional were apparent when discussing the importance of relationship development between all stakeholders.

Because you’ve got to trust them and they’ve got to trust, I suppose, a little bit in you as well. So, it’s nice to have that but they also know when to keep it professional, and when to keep it personal as well.

[02C]

Technical skills and animal handling capabilities were important to all clients and to those professionals with direct hands-on work as part of the day-to-day role.

If there’s a problem with a cow, and shall we say it’s what I would class as an internal problem where I can’t see any physical problems with the cow, obviously, I trust that the vet is able to make a good diagnosis.

[02C]

Trust and integrity were also expressed as a judgement on value for money.

Well because you are paying for that professional service and their opinions and that I’m entrusting them with the care of my animals.

[13C]

#### 3.1.4. Theme Four: Value for Money

Value for money, with price paid reflecting the service received, was an enduring theme throughout the interviews. Interestingly, veterinarians discussed financial implications more frequently than the other stakeholders, stressing the problems associated with a pricing strategy which does not reflect value.

At the moment, veterinarians haven’t been very good at charging for time, they’ve subsidized it by sales and medicine. That’s tempered the whole best way forward. The best way forward in my view is for veterinarians to sell their time and not much else.

[03V]

This was further emphasised when discussing farm animal practice and concepts of value related to price were introduced.

The vast majority of farmers have a high level of expectation of the vets. They’ve an expectation of good service, expectation of reasonable prices, but they know that they’re always going to get a reasonable sized total bill at the end of the month. That’s what they expect from vets. But they expect the highest standards and that’s okay as long as they can see the value.

[04V]

*Cost plays an element, but what we find is there are competitors in our area who would sell some wormers cheaper than us. But having spent an awful lot of time training people, our SQPs* [Suitably Qualified Persons/Animal Medicines Advisor]*, and the relationship we’ve built with clients, it’s not always about the price anymore.*

[04V]

Clients introduced the concept of involvement and preparedness to pay more money in situations which they perceived to have higher stakes, carry greater risk to the animal involved, or require higher levels of skill or technical ability from the professional.

For example, paying a full call out for them to come and do vaccinations, which they can do standing on their head. It doesn’t really take a lot of ability. But I think largely, considering what they’re doing, which is highly technical, I do think it is good value for money, knowing what similar things cost in medicine.

[07C]

#### 3.1.5. Theme Five: Confident Relationships

All of the study participants discussed the importance of two-way communication, through a mutually respectful relationship. Veterinarians particularly identified with the need to make every effort to communicate with clients, emphasising the importance of communication within the service process.

You have to actually communicate with the owner in every possible available way and develop that ability.

[04V]

Allied practitioners described the client expectations of communication and also to be active in the service process.

*Communication is one of the key things that they [*clients*] definitely expect and a follow-up as well. They are making sure that not only are they making that initial contact with the owner about something but the follow-up after that.*

[09P]

When discussing how involved clients expected to be in the service process, one practitioner indicated client expectations of the process.

*Clients will be expecting it [*involvement*], and clients will be driving that they have that for their animal.*

[10P]

Clients sought open, respectful, and intelligent communication with the veterinarian and allied practitioners alike. All groups made references to the need for courteous interaction between themselves and the service provider, with some owners placing importance upon how they were addressed.

*Will they [*veterinarians*] communicate with me in a professional manner, but also not treating me like I don’t know anything at all?*

[07C]

This concept was taken further by one client, who actively sought a challenging dynamic with the veterinarian and allied practitioners to ensure the best possible outcome for their livestock.

I have to say we’re very lucky with the people that we work with. They do challenge you. We possibly hopefully challenge them a little bit. We bounce ideas off each other. As I say they’ll often have meetings with our nutritionist, with the vet, and they’ll all sit down every couple of months together… It’s nice if they come out and give you ideas and suggestions, and challenge your thinking as well…

[12C]

Also, clients did not want their own personal experience to be discounted, seeking a personal involvement within the service process. This involvement was not distinct to a single client group, but was apparent through the companion animal owners, horse owners, and livestock farmers alike.

I don’t think a lot of veterinarians value the opinion of the owner, despite the fact that some owners are very experienced with their own horse or with a number of horses.

[07C]

Clients often drew on their human health experiences of communication to evaluate the veterinarian or allied practitioner.

*Because I think doctors are now taught to communicate. They do loads of role-play, especially if they’re going to be a GP* [General Practitioner Doctor], and realise, “Actually, I can communicate with these people, and it should be a two-way street. But I think people need to be taught to communicate. If you’re a four A* student, who has studied really hard, you may not have the social skills, the interpersonal skills. You need to learn those if you don’t have them naturally, which some people do.

[07C]

Professional interactivity indicative of co-creating and co-producing service was equally evident.

*I’m looking for them* [allied practitioners] *to be able to identify—obviously they have a conversation with me first about what my thoughts are. I think it’s important that I feel involved.*

[08C]

Clients introduced the concept of self-care to explain their desire for an active involvement and participation in the service process.

*She looked at how he* [the horse] *moved. She did a really in-depth assessment when she first met the horse, and then treated it very thoroughly, gave me exercises that I could do… I was very involved. I always want to feel that I can do my bit as well, and I can’t believe that somehow something just needs treating in three months, six months, twelve months. There’s got to be some kind of self-care in the meantime.*

[07C]

*So, it’s more listening to what the farmer wants, based on the herd size, and how they want to run things. It’s not just what they* [the vet] *think. It’s the involvement of the farmer and what he wants with his animals.*

[12C]

#### 3.1.6. Theme Six: Accessibility

Accessibility, physical and communication, emerged as a practical but important dimension which had relevance to all participants.

It is problematic. Just the thought that you can’t just get the vet when you want them is problematic to me, and booking so far in advance.

[02C]

It was two miles from where I lived, could always get an appointment straight away. I was always very pleased with the care that I got for all of my animals.

[13C]

The concept of accessibility through virtual communication was raised by an allied practitioner during the discussion of social media trends and the need for flexibility in communication techniques.

Enquiries on our advice line are actually dropping, and enquiries through social media are going through the roof. She’ll get a tweet at ten o ‘clock at night and answer it.

[05P]

Out of hours’ care and emergency care were crucial topics to the client group who keenly felt the importance of being able to contact the professional with ease and speed. 

## 4. Discussion

This study used a qualitative approach to explore relationship-centred care within the animal health provision from the perspective of the three core stakeholders, veterinarians, allied practitioners, and clients. The findings provide novel insight into the conceptualisation of co-produced service for the sector. Co-produced service is delineated by equal and reciprocal relationship development through a responsive service provision. Co-production has relevance and application to a diverse range of sectors, and results from this study propose applicability and potential practical implications for contemporary veterinary and animal health care. 

Within service quality provision, all service is assumed to be inherently relational in nature [45] as the client is endogenous to, and is an active participant in the service provided [46,47]. Within this study, concepts of trust, bonds, empathy, communication, and relationships were evidenced to be important components of the service experience. These findings reflect conceptualisation of value-creation in service and co-production [48] and correspond to previous studies completed on veterinarian–client interaction [20,21,22,49]. 

Confidence in relationships and relationship-centred care emerged as important to all stakeholder participants, but the theme was particularly well developed within the client group. Central to the strong development of partnerships between clients and practitioners was reciprocity, with emphasis given to two-way, respectful communication, similar to the findings of Coe et al. [49]. When discussing factors of trust, clients were keen to emphasise its importance but also to stress the reciprocal nature of trust between the client and professional. Trust is essential for collaborative working and co-production and is founded in the expectation that one party will behave in a predictable and reliable manner [50]. Trust may take a number of forms. Newell and Swan [51] determined three types of trust pertinent to collaborative working: companion trust, competence trust, and commitment trust. Companion trust is based on the reciprocal exchange of goodwill and friendship. Competence trust is established through perceptions of others’ ability to perform the required tasks. Commitment trust is associated with contractual arrangements or expectations between the clients and practitioner. Trust, in the medical, and arguably veterinary setting, is conceptually difficult to define and there is no commonly shared understanding of what it means, what factors affect trust or how it relates across the health provision [52]. In co-produced service, the veterinarian or practitioner must fully recognise the integral role of the client in the service process and, therefore, trust the clients’ judgement. 

Introduction of the notion of self-care was raised by the equine and farm animal clients within this study, reflecting their wish to be an active participant in the service process. The idea of involvement was a common feature to all client groups as they did not wish the value of their own personal experience to be ignored or dismissed, or to feel that they were not active in the health care process. Reciprocity of engagement and involvement was an interesting feature and novel to the client; to the farm animal client, this notion was so strongly developed that they expressed a wish to be ‘challenged by the professional’. Active participation in the service encounter is wanted by the client, a concept indicated by only a limited number of previous studies [23,49]. 

Conceptually, co-production extends inter-relationships beyond involvement into active partnerships, creating opportunity and challenges for veterinary service provision in equal measures. Client participants from the present study expressed their wish to be part of the service delivery. Every service encounter contributes to relationship formation [47,53] as co-produced service has the potential to improve the longevity of relationships [47,54] whilst synchronously building loyalty [55]. Loyalty in veterinary and animal health practice confers benefits of enhanced treatment outcomes and business sustainability. 

Authenticity of collaborations is central to relationship-centred care [56] and is reflected in this study through experiences of empathetic care. In analogous human health care, empathy is viewed as the cornerstone of the patient–medic relationship [57]. Yet, in the health context, clinical empathy is complex and problematic to describe, as protective mechanisms need to be in place to safeguard the medical practitioner from repeated exposure to often upsetting scenarios. The potential for conflict is apparent, as patients desire true, authentic empathy, whilst practitioners may need to maintain clinical detachment to safeguard their own health [58]. Given similarities in the roles performed, it could be assumed that the veterinarian or allied practitioner would experience the same tension and this was confirmed as client participants from the present study expressed the value they placed on true empathy. 

Factors of continuity of care were raised equally by all stakeholder contributors, emphasising the value of relationship formation between client and practitioner. In human health service, continuity of care and the development of strong relationships between the patient and medical practitioner are known to improve service satisfaction [59], treatment adherence, and outcomes [60,61]. Where sustained continuity of care is present, communication between the patient and physician is enhanced and service satisfaction improved. The nature of allied practitioner service in the animal health sector often facilitates continuity through repeated service encounters with the same clients. Conversely, in contemporary veterinary practice organisations, this can be difficult to achieve and now presents as a sector challenge. 

At the policy level in human health practice, service–user collaboration is an accepted requirement [30,31] and the patient is an active participant. This is not without challenge. Challenges in human health services are cited as: external performance pressures, professional norms and values, and culture [30,31]. They serve as functional barriers to the inclusion of the patient and make client participation a complex offering. Findings from the present study strongly indicate stakeholder expectations of a co-produced service, but practitioner acceptance of the client as an active service collaborator raises thought-provoking questions for daily practice. The paucity of evidence on animal health client service expectations make framing a co-produced service for the sector challenging. A co-produced approach can be complex, nuanced, and intricate [62], but quality of communication and trust are central. There must be mutual acceptance of the partnership between clients and the veterinarian or practitioner in order for co-produced service to be delivered. The present study demonstrates that communication can serve as a proxy for trust, but where communication barriers exist or there is a failure in reciprocity of communication, co-produced service cannot be delivered [36]. 

**Figure 1:**
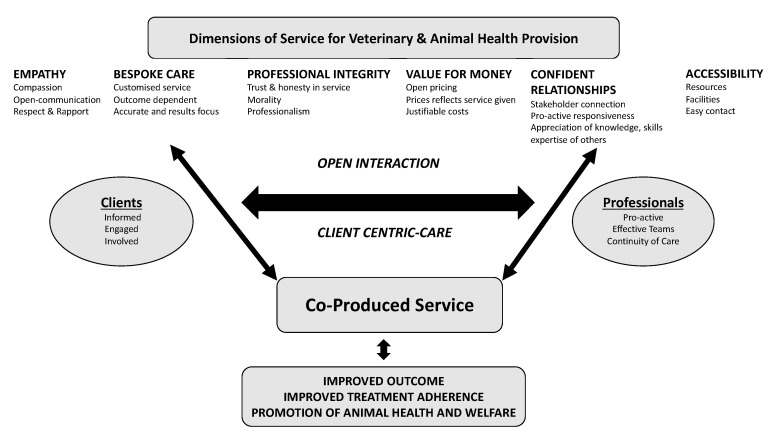
Dimensions of service for veterinary and animal health provision.

Results from the present study indicate that the provision of client-centred co-produced care requires a re-examination of existing practice, potentially a paradigm shift in service provision. Recommendations from human practice for co-produced service indicate the requirement for: involvement of patients in decision-making processes, patient-centred tailored care with a move away from standardised protocols, and a shift in power-dynamics as the patient takes more control of the health care delivery [35,37]. These recommendations create a starting point for developing our understanding of co-produced care contextualised for the veterinary and animal health sector, as do the findings from the present study. A framework to illustrate a co-produced framework for the sector is presented in Figure 1.

It is evident from the stakeholder interviews that co-production is integral to service quality and service satisfaction, and accordingly, is under active research. Lacking is pragmatic research into how to implement co-production from a day-to-day management perspective. It is not always clear what constitutes co-production and defining co-production or what is being co-produced is subject to discussion [63], with the proposition that there could be multiple versions of co-production contextualised to the sector or situation.

**Figure 2:**
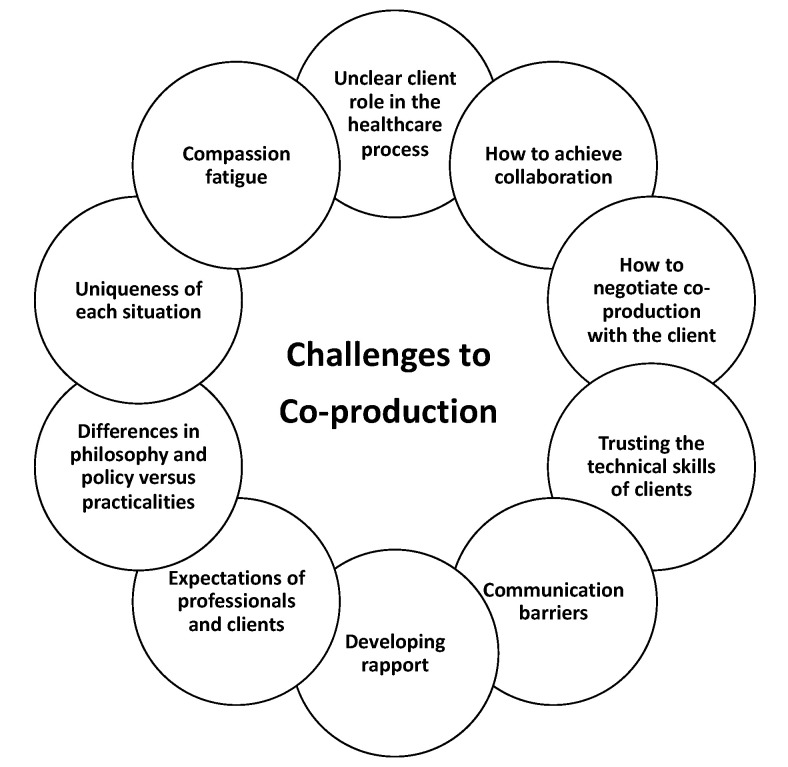
Challenges to co-production.

The proposed navigational challenges contextualised for the veterinary and animal health sector, as determined by this study, are presented in Figure 2. Reflected are diversity and variation, and the differences in meaning and scope for co-production. Irrespective of the complexities of co-production, it is transparent that the quality of relationships permits co-production [64] and is evidenced through this study. Co-produced veterinary service requires a significant shift of power, as it moves beyond straightforward involvement of the client, to the establishment of equal and reciprocal partnerships. Flexibility in resources and time and blurring of practitioner–client boundaries [56] are cited as requirements for effective co-produced care. As a people-centric framework and a relationship-centred approach, co-production accepts health care recipients as active participants in their care [65], conferring benefits of enhanced health service efficiency as those who use the service are valuable resources [32]. Recognition by clients and professional alike of bespoke service was confirmed in the present study, supporting anecdotal indications that clients’ expectations of service will continue to rise. 

Hamilton’s 2018 [66] review of adopting a co-creative approach to our understanding of the vet–farmer relationship, proposes co-production as an alternative to the evidence-based methodology most frequently adopted for vet–client communication research. Development of veterinarian–farmer partnerships has been marginally explored [67]. Integration of the client in an active, reciprocal partnership requires a significant forward leap in veterinary care but may reflect the future of animal health service provision. 

## 5. Further Work

As far as the authors are aware, this is the first study to examine co-production for veterinary service provision. It is accepted that there are study limitations and equally, many questions for further investigation have arisen. However, research into allied health services in the veterinary domain is often over-looked, irrespective that these professionals are integral to the vet-led team and the overall service provided. A more enhanced balance in interview participants could have been achieved with the inclusion of more veterinarians (including equine practitioners), and the broad study approach taken leads to generalised results. Thus, further work to clarify differences in service provision between different allied practitioners and veterinarians would be of value.

As a novel work, the study outputs raised many areas for future research. Fundamental questions are raised on our understanding of how to provide co-produced care across animal health and veterinary services and how this may effectively be integrated into daily practice. Questions on the applicability of co-production across distinct animal health sectors (farm animal, equine, and companion animal) within the UK have been raised, and also across international veterinary services and practice. This study has highlighted the potential practical barriers for co-produced service, but these require further investigation and evaluation to understand the challenges from the perspective of different practitioners and sub-sections of the veterinary sector.

Whilst benefits of enhanced relationship development between client and practitioner through co-produced care are evident, the impact of prolonged authentic care on practitioner compassion fatigue and resultant work-related stress remain to be fully understood.

## 6. Conclusions

The co-produced nature of services is a well-developed concept across the services sector and is proposed and supported through the results of the present study to be relevant and valuable to the veterinary and animal health services. The emergent dimensions are strong advocates for the wider adoption of a co-produced service for the sector, but equally, the pragmatic challenges are identified. Quality communication serves as a proxy for relationship formation and could aid the development of co-produced service provision from practitioners. Client involvement in the animal health care process is evident through the stated wish for active participation in the service process and a strong desire for reciprocity of communication delivered through a robust practitioner–client relationship.

## Figures and Tables

**Table 1:** Summary of participating stakeholders (*n* = 13) by gender, age, and stakeholder type.

Code	Classification	Gender	Age (Approximate)	Occupation	Additional Information
01P	Allied Practitioners	F	60	Musculoskeletal professional	Equine specialist
02C	Client	M	35	Farmer	Extensive mixed livestock
03V	Veterinarian	M	68	Farm and mixed practice	Industry knowledge transfer
04V	Veterinarian	M	50	Farm	Referral mixed practice
05P	Allied Practitioners	F	40	Senior nutritionist	Equine specialist
06P	Allied Practitioners	M	70	Veterinary pharmacist	Companion animal specialist
07C	Client	F	50	Medical writer	Dog and horse owner
08C	Client	F	30	Dog trainer	Dog and horse owner
09P	Allied Practitioners	F	20	Veterinary nurse	Mixed practice
10P	Allied Practitioners	M	30	Musculoskeletal practitioner	Equine specialist
11V	Veterinarian	F	30	Companion animal specialist	Charity companion animal
12C	Client	F	30	Farmer	Intensive dairy
13C	Client	F	50	Administrator	Dog owner

**Table 2:** Definitions of the dimensions of service identified from the interviews.

Theme	Definition
Empathy	Compassion and thoughtfulness through a clearly communicated service. Caring provision, with due regard for clients’ needs and animal health and welfare.
Bespoke Care	Custom tailored, dependable service which is accurate and a results-focused provision.
Professional Integrity	Trust, honesty and morality of service delivery. Strong themes of professionalism.
Value for Money	Willingness to provide comprehensive service within a justifiable pricing strategy. Price paid reflects the service given.
Confident Relationships	Professionals’ connection with the client, connection with other professionals, and pro-active responsiveness to the wider knowledge, skills, and expertise of others. Preparedness to undertake two-way open communication with an active client, demonstrative of respect and rapport.
Accessibility	Geographical proximity of up-to-date resources and facilities, accessibility of professionals (physical and communicative), and ease of contact.

## Supplementary Materials

The following are available online at https://www.mdpi.com/2306-7381/7/4/149/s1.
