# Supplementary material for: Co-Produced Care in Veterinary Services: A Qualitative Study of UK Stakeholders’ Perspectives
Source: Vet Sci. 2020 Oct 1;7(4):149. doi: 10.3390/vetsci7040149 (PMC7712434; doi:10.3390/vetsci7040149)
Supplement: Supplementary file 1 [file vetsci-07-00149-s001.pdf]

Co-produced veterinary services

Interview questions- Client version (veterinary services)

**Question**	**Probing/ prompts questions**<br>**Questioning technique**
Opening question:<br>Tell me a bit about yourself, your profession, your animals, and your experience of keeping animals.	
**Veterinarian Service Questions**	
Tell me about the veterinarian that you currently use.	What do you use them for ?<br>Have you used them for a long time?<br>How did you find them/ select them?<br>Recommendations/ advocates.
What qualities are you looking for in the veterinarian?	Personal qualities.<br>Price.<br>Location.<br>Specialism.
When did you last have the veterinarian out and why?	How easy was it to make the appointment?<br>Talk me through the appointment / process.
What, if any, expectations do you have of the veterinarian before the appointment / or their arrival ?	Practitioner personal qualities.<br>Appearance.<br>Communication.
What, if any, expectations do you have of the service beforehand?	Open questioning
How do you know that they have done a good job?	Do they ever ask you if you are happy with the service they provide?<br>How do you rate the service they offer?
Do you feel that you get value for money?	Can you outline your thoughts ?<br>Why do you feel this way?
Has there ever been an occasion when a treatment has been unsuccessful?	*Critical incident discussion*<br>What happened?<br>Why have you felt let down?<br>Have you ever sought a 2nd opinion? What may make you seek a 2nd opinion?
Has there ever been an occasion when a treatment has surpassed all of your expectations?	*Critical incident discussion*<br>What happened?<br>Can you explain why you feel this way?<br>What did the veterinarian do to make you feel this way?

Interview questions- Client version (allied animal health services)

Question	Probing/ prompts questions<br>Questioning technique
**Allied Animal Health Practitioner Service Questions**	
Tell me about the/an allied animal health practitioner that you currently use.	What do you use them for ?<br>Have you used them for a long time?<br>How did you find them/ select them?<br>Recommendations/ advocates.
What qualities are you looking for in the allied animal health practitioner ?<br>NB Clients may wish to refer to a specific group of practitioners.	Personal qualities.<br>Price.<br>Location.<br>Specialism.
When did you last have the allied animal health practitioner out and why?	How easy was it to make the appointment?<br>Talk me through the appointment / process?
What, if any, expectations do you have of the allied animal health practitioner before the appointment / or their arrival ?	Practitioner personal qualities.<br>Appearance.
What, if any, expectations do you have of the service beforehand.	Open questioning
How do you know that they have done a good job?	Do they ever ask you if you are happy with the service they provide?<br>How do you rate the service they offer?
Do you feel that you get value for money?	Can you outline your thoughts ?<br>Why do you feel this way?
Has there ever been an occasion when a treatment has been unsuccessful?	*Critical incident discussion*<br>What happened?<br>Can you explain why you feel this way?<br>What did the allied animal health practitioner do to make you feel this way?
Has there ever been an occasion when a treatment has surpassed all of your expectations?	*Critical incident discussion*<br>What happened?<br>Can you explain why you feel this way?<br>What did the allied animal health practitioner do to make you feel this way?
**Team working questions**<br>Has there ever been an occasion when the vet has worked with the allied animal health practitioner?	How did that work out for you?<br>What was the service like?<br>Was it easy to organise?
**Closing Question**<br>Is there anything that you would like to add which you feel we have not covered today but is relevant to the service that you have received?	

Interview questions – Professional version

Question	Probing/ prompts questions<br>Questioning technique
Opening question:<br>Tell me a bit about yourself, your training, and your profession.	
**Your Clients**	
Tell me about your client base.	Who are your clients?<br>Describe your typical client.<br>How many clients do you regularly see?<br>Is the client base loyal?
What do you think your clients want from your service?	How you know what your clients want?<br>Do you meet these needs?
What expectations do you think that your client has of you as a professional?	Where appropriate, does this vary for different groups of clients?
What personal qualities do you think the client seeks in the veterinarian/ allied animal health practitioner?	What makes you think that those qualities are important?
Have you had a situation where an appointment/consultation/ treatment has not worked out or gone to plan?	Critical incident discussion<br>What happened?<br>How did you feel? How did the client feel/respond?<br>What was the outcome ?
Have you had a situation where a treatment/ appointment worked particularly well?	Critical incident discussion<br>What happened?<br>How did you feel? How did the client feel/respond?<br>What was the outcome ?
How do you provide a quality service?	What is service quality in your profession?
How closely matched is your service to what you perceive to the ideal?	Why do you hold that opinion?
In your profession, tell me how you feel the services have changed?	Client expectations.<br>Changed in a positive or negative way.<br>Sustainable change.
**Team working questions**<br>In your daily role do you work with specialists colleagues (interdisciplinary working)	How does this work out for you?<br>If there are any omissions why don't you work with this group?<br>How easy do find referring clients to other professionals?
**Closing Question**<br>Is there anything that you would like to add which you feel we have not covered today but is relevant to the service you provide?	
